# Impact of Zinc, Vitamins C and D on Disease Prognosis among Patients with COVID-19 in Bangladesh: A Cross-Sectional Study

**DOI:** 10.3390/nu14235029

**Published:** 2022-11-26

**Authors:** Nadim Sharif, Rubayet Rayhan Opu, Afsana Khan, Khalid J. Alzahrani, Hamsa Jameel Banjer, Fuad M. Alzahrani, Nusaira Haque, Shahriar Khan, Saimum Tahreef Soumik, Ming Zhang, Hanwen Huang, Xiao Song, Anowar Khasru Parvez, Shuvra Kanti Dey

**Affiliations:** 1 Department of Microbiology, Jahangirnagar University, Dhaka 1342, Bangladesh; 2 Department of Statistics, Jahangirnagar University, Dhaka 1342, Bangladesh; 3 Department of Clinical Laboratories Sciences, College of Applied Medical Sciences, Taif University, Taif 21944, Saudi Arabia; 4 Department of Epidemiology and Biostatistics, College of Public Health, University of Georgia, Athens, GA 30602, USA

**Keywords:** vitamin C, vitamin D, zinc, COVID-19, treatment, Bangladesh

## Abstract

Vitamin C, (ascorbic acid), vitamin D (cholecalciferol) and zinc (zinc sulfate monohydrate) supplements are important in immunity against coronavirus disease-2019 (COVID-19). However, a limited number of studies have been conducted on the association of vitamins and supplements with the reduced risks of COVID-19 infection. This study aims to evaluate the association of vitamins and supplements as treatment options to reduce the severity of COVID-19. Data were collected from 962 participants from 13 December 2020 to 4 February 2021. The presence of COVID-19 was confirmed by qRT-PCR. The Chi-square test and multivariate regression analyses were conducted. The ratio of uptake of vitamin C:vitamin D:zinc was 1:1:0.95. Uptake of vitamin C, vitamin D and zinc were significantly associated with the reduced risk of infection and severity of COVID-19 (OR: 0.006 (95% CI: 0.03–0.11) (*p* = 0.004)) and (OR: 0.03 (95% CI: 0.01–0.22) (*p* = 0.005)). The tendency of taking supplements was associated with the presence of infection of COVID-19 (*p* = 0.001), age (*p* = 0.02), sex (*p* = 0.05) and residence (*p* = 0.04). The duration of supplementation and medication was significantly associated with reduced hospitalization (*p* = 0.0001). Vitamins C, D and zinc were not significantly (*p* = 0.9) associated with a reduced risk of severity when taken through the diet. Hospitalization (*p* = 0.000001) and access to health facilities (*p* = 0.0097) were significantly associated with the survival period of the participants. Participants with better access to health facilities recovered early (OR: 6.21, 95% CI 1.56–24.7). This study will add knowledge in the field of treatment of COVID-19 by using vitamins and zinc supplements.

## 1. Introduction

The COVID-19 pandemic is shattering incidence records globally [1,2]. In Bangladesh, at least 1.6 million cases and 28,000 deaths have been documented [3,4]. Bangladesh is a densely populated country (1265 Person/Km^2^) with a total population of 164.7 million in 2021. About 9674 persons have been infected and 168 persons have died of COVID-19 per million people in Bangladesh [3,4]. Male patients aged above 40 years were more likely to be infected by COVID-19 and the death rate was also high among them [1,2,3,4,5].

Severe acute respiratory syndrome coronavirus-2 (SARS-CoV-2) is a (+) sense RNA virus. It was named after the crown-shaped spikes on the pathogen surface [5,6]. SARS-CoV-2 causes COVID-19, which displays a wide range of clinical symptoms, including asymptomatic infection, moderate upper respiratory tract infection, and severe pneumonia with respiratory failure, which can lead to hospitalization requiring intensive or sub-intensive care [2,7,8]. The nutritional status of individuals can influence the proliferation of different blood cells associated with immunity [9]. The role of dietary supplementation of vitamins and micronutrients in stimulating specific immunity against COVID-19 needs to be explored in detail [10]. However, taking Vitamin C, Vitamin D and zinc as medicines with specific doses may help reduce nutritional gaps, support optimal immune functions, and possibly reduce the risk and consequence of infection of COVID-19 [11,12,13,14].

Vitamin C (ascorbic acid) is a water-soluble micronutrient with antioxidant properties that readily acts as a one or two-electron reducing agent for free radicals and oxidants [15,16]. It plays a pivotal role in the immune system by supporting the epithelial barrier against the entry of pathogens and the cellular functions of the innate and adaptive immune systems [16,17]. There is also evidence that vitamin C may improve the health status of patients with pneumonia and infection owing to its direct inhibitory effects on pathogens [11,16,17]. Vitamin C may be an adjuvant to acute respiratory distress syndrome (ARDS). It may lessen the negative effects of sepsis associated with acute respiratory dysfunction, and lower the incidence of pneumonia by 80% [16,17,18]. The administration of vitamin C in a high oral dose (60 mg/kg) enhanced natural killer cell activity, which plays an important role in innate immunity against viral infection [16,17,18,19].

Vitamin D (cholecalciferol) is an essential lipid-soluble vitamin, produced endogenously with the effect of ultraviolet radiation on the skin or available from exogenous food sources or dietary supplements [20]. Further, vitamin D modulates the function of immune cells, such as T and B cells, monocytes and dendritic cells, which interact between the innate and adaptive immune systems [20,21,22]. Vitamin D decreases the expression of pro-inflammatory cytokines, such as tumor necrosis factor and interferon-gamma (INF), and increases the expression of anti-inflammatory cytokines and also promotes the induction of T regulatory cells, thereby inhibiting inflammatory processes [13,19,20,21,22].

Zinc (Zn) is the second most abundant trace metal in the human body after iron [23]. Zinc has regulatory effects on the growth and activity of T cells and the cytokine storm [23,24]. Zinc deficiency reduces the function of natural killer (NK) cells and cytolytic T cells, both of which help to destroy viruses, bacteria and tumor cells [23,24,25].

There are not enough studies focusing on the effects of micronutrients and trace elements in reducing the severity of the COVID-19 pandemic. A few studies with a small number of samples and clinical trials are available on the beneficiary effects of taking vitamin C, vitamin D and zinc (zinc sulfate monohydrate) as medicine for stimulating the immune system to fight COVID-19. Epidemiological studies assessing the impact of vitamins and minerals in reducing the risk of infection and fatality associated with COVID-19 are required to predict more effective treatment options. Therefore, this study was conducted to determine the association and impact of vitamin C, vitamin D and zinc as medicine on reducing the risks of fatalities and infection of COVID-19 among Bangladeshi people. The main aim of this study was to assess the impact of vitamins and minerals taken as medicines on the reduction in risks of infection and severity of COVID-19. Another aim was to evaluate the impact of dietary supplementation on the COVID-19 pandemic.

## 2. Materials and Methods

### 2.1. Ethical Approval

The ethical clearance was obtained from the Biosafety, Biosecurity and Ethical Committee at the Jahangirnagar University. The ethical approval number of this study is BBEC, JU/M 2021/COVID-19/(1)2. Informed consent was taken from the participants before taking the survey.

### 2.2. Study Area and Population

During the COVID-19 pandemic, a cross-sectional study was conducted from 13 December 2020 to 4 February 2021 across 35 districts in Bangladesh. About 962 people participated in this study. Data were collected from each individual. COVID-19-positive cases were confirmed by real-time quantitative reverse transcription polymerase chain reaction (qRT-PCR). Data from children aged below 16 years were collected from their parents. Informed consent was taken from the parents before enrolling in this study. Data on the dead patients were taken from their family members. Inclusion criteria were as follows: participants taking any of the vitamins or minerals or a combination of both vitamins and minerals, participants testing positive for COVID-19 in qRT-PCR, participants showing symptoms of COVID-19 and having one or more family member positive for COVID-19, participants staying at the residence city for longer than one month, and participants not having other severe health conditions or history of taking other medication that might interact with the uptake of vitamins and minerals. The exclusion criteria included participants giving incomplete information, participants taking irregular vitamins and minerals, participants not tested by qRT-PCR or any of their family members not confirmed by qRT-PCR, and participants being able to show their travel history within the last 30 days. About 27 participants were excluded from this study due to incomplete data.

### 2.3. Data collection Tools and Variables

A standard and validated questionnaire was developed and used for data collection from the participants. The questionnaire was developed in English language and translated into the participants’ native language (Bengali) and administered online. The questionnaire included variables on socio-demographics, COVID-19 test status, COVID-19-related health status, symptoms, medication status, vitamin C, vitamin D, and supplementary zinc uptake history among infected and healthy participants before and after COVID-19 infection, and COVID-19 recovery time. Socio-demographic variables included age, sex, occupation, family income, current place of residence (village, district town and divisional town), and access to health facilities. Research assistants (Ras) circulated and collected information from the participants. About 20 Ras were involved in this study for data collection.

The factors taken into consideration were whether the participants consumed any of the vitamin C, vitamin D and zinc through supplements, regular diets or both, the duration of consumption, and the amount consumed.

### 2.4. Statistical Analysis

Appropriate statistical analyses were conducted to determine the association of vitamins and supplementary diet with the infection and fatality of COVID-19 in this study. Continuous data were presented as mean and standard deviation (SD), and categorical data as frequency and percentage.

The Chi-square test was conducted to assess the association between socio-demographic factors and the tendency to uptake medication and vitamin supplementation during COVID-19. The logistic regression model was used to study the impact of vitamins, minerals, medicines and host factors on the COVID-19 infection and severe outcome. Both the vitamins taken as medicine and taken from diets were analyzed and compared. Sex, hospitalization, and access to health facilities were included as confounding variables.

Further, the ordinal regression model was applied to study the influence of vitamins on the COVID-19 survival time in COVID-19 participants. Three ordered categories were considered: 1–5 days, 7–14 days and 15 or above days. Both the vitamins as ingredients from medicines and food were analyzed. Sex, hospitalization and access to health facilities were included as confounding variables. The standard statistical inference tool was used to determine the significant associations between categorical dependent and independent variables by controlling the type-I error (*p*-value) at a 5% level. All statistical analyses were performed by using Stata software version 17 (StataCorp, College Station, TX, USA).

## 3. Results

### 3.1. Socio-Demographic Characteristics and Distribution of the Participants

**Figure 1:**
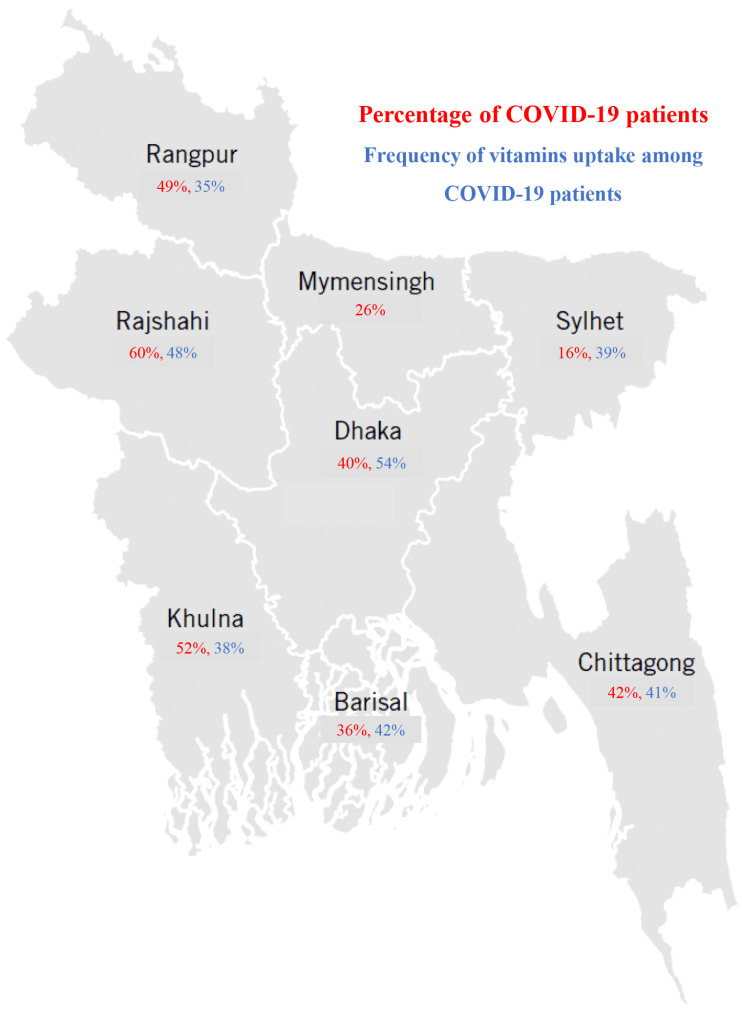
Nationwide frequency distribution of participants and COVID-19 patients.

Among 962 participants, the ratio of male/female was 2.08. The participants were divided into eight age groups, including 5–9, 10–19, 20–29, 30–39, 40–49, 50–59, 60–69 and above 70 years. The mean age of the study population was 34 ± 4.6 years. Most of the participants belonged to the age group 20–29 years (40%), followed by 30–39 years (19%) and 40–49 years (16%), respectively (Table 1). The highest frequency of the participants was students (33%) with no monthly income (45%). About 79% (757 of 962) of participants were from divisional cities, and a majority of them (79%, 757 of 962) had better access to health facilities (Table 1). The participants were distributed in 35 districts in Bangladesh. Among the eight divisional cities, most of the study population was from Dhaka (32%), followed by Chittagong (23%), Rajshahi (17%), Khulna (14%) and Sylhet (6%), respectively. Among 962 study participants, 503 (52%) were positive for COVID-19. Most of the positive cases were reported from Dhaka (43%), followed by Rajshahi (27%) and Chittagong (23%), respectively (Figure 1).

### 3.2. Frequency Distribution of Vitamins and Supplements among COVID-19-Positive Participants

About 52% (503 of 962) of the participants were positive for COVID-19 infection by qRT-PCR and the remaining 459 participants (48%) were suspected cases of COVID-19. Suspected participants had close contact with one or more COVID-19 positive family members. The severity of symptoms varied among them. The study participants were divided into two groups based on the source of vitamins and nutrient uptake. One group took vitamins and nutrients from medicine tablets and another group took vitamins and nutrients from food items. In Table 2 we included the participants who took vitamins and nutrients from medicine tablets. Among the positive cases, 63% were male, and 37% were female. About 50% of the positive patients took one or more medicines among paracetamol, fexofenadine, and montelukast. About 10% (50 of 503) of the positive population took remdesivir along with these medicines (Table 2). Vitamin C was taken by 55%, vitamin D by 55%, zinc by 47%, and calcium by 47% of the positive participants. The frequency of taking vitamins and supplements was highest (~50 to 68%) among people aged 20 to 59 (Table 2). Fever was the most common symptom (74%, 375 of 503), followed by dry cough (36%, 182 of 503), loss of smell and test (34%, 173 of 503), body aches (27%, 137 of 503), fatigue (23%, 114 of 503), shortness of breath (19%, 98 of 503), sore throat (17%, 88 of 503) and diarrhea (7%, 37 of 503), respectively. The symptoms prevailed for 7–14 days among 70% of the patients and more than one month in only 4% of the patients. The highest frequency of severity of symptoms was mild (89%), followed by severe symptoms (10%). The symptoms resolved among 32% of the patients within 5 days, 50% within 7 -14 days, and 14% within 15–30 days of taking vitamins, supplements and medicines (Table 2).

### 3.3. Association of Medicine and Vitamin Uptake with Socio-Demographic Factors

The Chi-square test was conducted to determine the association between the tendency to uptake the vitamins and the socio-demographic factors, residence and COVID-19 infection status. Age was significantly associated with the uptake of vitamin C (*p* = 0.04), vitamin D (*p* = 0.03) and zinc supplementation (*p* = 0.05) among the participants. Sex, occupation, monthly income and residence were also associated with the practice of taking vitamins and supplements among the participants (Table 3). Infection by COVID-19 of the participants was significantly associated with the uptake of medication (*p* = 0.001), vitamin C (*p* = 0.001), vitamin D (*p* = 0.05) and zinc supplements (*p* = 0.001). The presence of symptoms of COVID-19 and the uptake of vitamins and supplements were also significantly associated (Table 3).

### 3.4. Impact of Vitamins and Supplementation with COVID-19 Infection

The impacts of the increased as well as unbothered dietary and supplementary intakes of vitamins and minerals as medicines were both observed. Vitamin C was prescribed and taken as a vitamin C 500 mg tablet one time per day, vitamin D was taken as cholecalciferol (D3) 2000 IU single tablet per day, and zinc was taken as zinc sulfate monohydrate 20 mg single tablet per day. After controlling for the confounding variables (sex, hospitalization and health facility), the logistic regression analysis revealed that the uptake of vitamin C, vitamin D and zinc as medicine was significant to reduce the risk of infection among the participants (OR: 0.006, (95% CI: 0.03–0.11) (*p* = 0.004)). Vitamin uptake as medication was associated with the reduction in infection among the participants. However, increased uptake of vitamin C and vitamin D-enriched foods was not significantly associated with a reduced rate of infection (OR: 0.97 (95% CI: 0.51–1.85) (*p* = 0.09)) and (OR: 0.97 (95% CI: 0.51–1.85) (*p* = 0.09)), respectively (Table 4). The odds ratio for infection rate was (0.318 (95% CI: 0.22–0.441) (*p* = 0.005)) between vitamin-taking and not-taking participants. Moreover, males had a higher risk of infection than females (OR: 2.51 (95% CI: 1.21–4.67) (*p* = 0.03)). Taking medicine against COVID-19 was significantly associated with reduced odds of infection among the participants (OR: 0.07 (95% CI: 0.01–0.94) (*p* = 0.03)) (Table 4).

### 3.5. Impact of Vitamins and Supplementation on the Outcome of COVID-19

The ordinal regression analysis showed that participants aged above 40 years had significantly higher odds of developing severe health conditions due to COVID-19 infection (OR: 5.61 (95% CI: 2.91–7.14) (*p* =0.05)). Participants taking vitamin C, vitamin D and zinc as medication had significantly reduced odds of developing severe diseases (OR: 0.03 (95% CI: 0.01–0.22) (*p* =0.005)) (Table 5). Participants with increased uptake of vitamins and minerals through diet were not significantly related to the reduced severity of COVID-19 (Table 5). However, hospitalization and better access to health facilities had a significant impact on the reduced odds of the severity of COVID-19 (OR: 0.03 (95% CI: 0.01–0.6) (*p* = 0.007)). It took longer for hospitalized participants to recover compared with non-hospitalized people (OR: 0.328 (95% CI: 0.219–0.493) (*p* = 0.005)). This is because the hospitalized patients had more serious symptoms than the non-hospitalization patients. Participants who had access to better health facilities were more likely to take less time to recover compared with those who had poor access (OR: 6.21 (95% CI: 1.56–24.7) (*p* = 0.00001)).

## 4. Discussion

The COVID-19 pandemic has been transmitted throughout the majority of communities worldwide [3,4,5]. Treatments for reducing the severe conditions and symptoms associated with COVID-19 involve different combinations of medicines and supplements [26,27]. Among different supplemental medicines, vitamin C, vitamin D and zinc have been found effective in reducing symptoms of different flu-like diseases including COVID-19 [27,28,29,30,31,32,33,34,35,36]. The association of vitamin C, vitamin D and zinc supplementation with the reduced severity of COVID-19 is required to be assessed precisely in public health aspects.

This study found that the uptake of vitamin C, vitamin D and zinc as supplements was significantly associated with the reduced odds of infection rate (OR: 0.006, (95% CI: 0.03–0.11) (*p* = 0.004)) and severe outcome of COVID-19 (OR: 0.03 (95% CI: 0.01–0.22) (*p* =0.005)) among the participants. This association of vitamins and minerals with reduced severity of COVID-19 might be due to the effect on the immunity of the body, which had been proven in the previous studies. These findings are in good agreement with the previous studies in China, India, USA, UK and Russia [26,28,29,30,31]. However, we found that vitamins and mineral taken through diets were not significantly associated (*p* = 0.09 and *p* = 0.87, respectively) with the reduced severity of COVID-19. Eating foods enriched in these vitamins had no significant association with lowering the risks of infection. Findings from this study support previous works and add new insights to understand the impact of the application of supplementary medicines in treating COVID-19 [36,37,38,39,40,41,42,43,44,45,46]. This insignificant association of vitamins and mineral taken through different foods with COVID-19 might be due to less absorption during digestion, interaction with other nutrients and elements, and poor digestive systems. Further, in this study, we detected that the participants were prescribed and had taken vitamin C, vitamin D and zinc as supplements. During the panic situation, people with less to mild symptoms similar to COVID-19 had also taken supplementary vitamin C, vitamin D and zinc. About 55% of our study patients took vitamin C and vitamin D, and 47% took zinc as supplements, which is similar to the previous findings in USA, India and China [29,30,31,32].

About 55% of the COVID-19-positive participants took vitamin C (500 mg) as a chewable tablet once a day, a vitamin D (vitamin D3 2000 IU) tablet and 47% took zinc as zinc sulfate monohydrate (20 mg) per day. About 43% of them took vitamin C, vitamin D and zinc as supplements. Participants taking only vitamin C, only vitamin D, and only zinc also had reduced odds of infection by COVID-19 and reduced severity of the disease. Further, we also assessed the impact of different medicines on the severity of the disease among the participants. Among the prescribed and used medicines, paracetamol 500 mg, fexofenadine hydrochloride 120 mg, montelukast sodium 10 mg, remdesivir 200 mg and antibiotics of different groups were evaluated for their association with COVID-19. Medicines were significantly associated (OR: 0.01 (95% CI: 0.008–0.07) (*p* = 0.001)) with reduced odds of the severity of COVID-19 among the participants. These findings are supported by previous studies [27,28,29,30,31,32,33,34,35,36,41,42,43,44,45]. Further, not taking any medicine, only vitamins and minerals taken as a supplement, was also effectively associated with the reduction in severity (OR: 0.8 (95% CI: 0.3–1.9) (*p* = 0.005)) of COVID-19. Findings from this study are in good agreement with previous studies supporting that vitamins and minerals are associated with the improvement of health conditions of COVID-19-infected individuals [32,33,34,35,36,37,38,39,46,47,48,49]. Further, a study on the association of vitamin D with a reduced requirement of ICU, oxygen support and death number among hospitalized patients also supports our findings [49]. Recent studies on the role of vitamin D against COVID-19 have found various positive impacts [43,44,45,46,47,48,49,50,51] including a molecular mechanism of preventing COVID-19 [51], association with reduced risk of infection [45], prevention of infection and reduction in the severity of COVID-19 [45,48]. Other studies have found that both vitamin C and Zinc can also contribute to the prevention and treatment of COVID-19 [45,50,51]. Our findings are highly similar to these findings. However, we could not determine the exact mechanism and preventive roles of these vitamins and nutrients. In the future, controlled studies on the preventive roles of vitamins and minerals in larger samples will be able to predict the association clearly.

Further, better access to health facilities reduced the risks of fatality among the participants significantly (*p* = 0.0097). These findings are in good agreement with previous works in Bangladesh and France [7,8,32]. Two previous studies in Bangladesh were of similar design and the findings on the association of better access to health facilities with reduced risks of fatality were also represented in our study [7,8]. The other study on the association of vitamin D with better survival of elderly patients with COVID-19 in France was a Quasi-Experimental Study. This study presumed that the supplementation of vitamin D was associated with better survival in elderly patients. However, in this study, we also included different types of medicines with vitamins and supplements prescribed to reduce symptoms in patients.

The frequency of male cases was (63%) higher than female, which is similar to previous works in Bangladesh [7,8]. The symptoms prevailed for 7–14 days among 70% of the patients. Fever was the most common symptom (74%, 375 of 503) followed by dry cough (36%, 182 of 503), loss of smell and taste (34%, 173 of 503) and body aches (27%, 137 of 503), respectively. These findings are in good agreement with previous epidemiological studies in Bangladesh [2,7,8]. Fatality was reported in only 2% (11 of 503) of the patients, which is lower than previous findings in Bangladesh [7,8]. Those studies did not report the inclusion of vitamin or medication effects, which might have impacted the outcome of the disease significantly and reduced the frequency of death among the patients.

We also analyzed the association between the socio-demographic factors and COVID-19 infection status with the tendency of taking vitamins and supplements among the participants. This study detected a significant association between the socio-demographic factors with the tendency of taking supplements as treatment options. Infection by COVID-19 of the participants and the presence of symptoms were significantly associated with the uptake of vitamin C, vitamin D and zinc supplements. Our study showed that vitamin C, vitamin D and zinc were supplemented with regular medicines for COVID-19 patients, which is in good agreement with the previous studies [3,14,27,28,29,30,45]. Age above 40 years (*p* = 0.0001), male participants (*p* = 0.03) and participants with higher family income (*p* = 0.0006) had significantly higher odds of infection. However, the odds of developing severe infection reduced (OR: 0.4 (95% CI: 0.1–1.9)) in participants with higher incomes. These findings are supported by previous cross-sectional studies in Bangladesh [7,8,46].

Other studies revealed that the progress of COVID-19 infection was associated with the increased production of C-reactive protein, pro-inflammatory cytokines, and increased risk of pneumonia, sepsis, acute respiratory distress syndrome and heart failure [34]. Inadequate nutrition and vitamins are contributing factors to the development of viral infection that weakens the immune system, which, in turn, increases the rate of infections and the risk of mortality and morbidity [15,22,23]. In the view of available contexts, there is robust evidence of the immunomodulation and anti-inflammatory activity of zinc and vitamins C and D, and so their deficiency, even if marginal, can compromise metabolism and, consequently, their action on the immune system [9,15,16,20,23]. Recently, a pilot study on 21 critical COVID-19 patients observed low serum levels of vitamin C and vitamin D among the patients [30]. In addition, older age and low vitamin C levels appeared to be co-dependent risk factors for mortality, suggesting that serum vitamin C levels contributed to the significance of age as a predictor of mortality [31]. A meta-analysis of 18 controlled clinical trials showed that oral or intravenous vitamin C reduces both the length of stay in the intensive care unit (ICU) by 7.8–8.6% (*p* = 0.003) and the duration of mechanical ventilation by 18.2% (*p* = 0.001) [34]. A high dose of vitamin C may be a proven therapeutic agent that not only ameliorates oxidative stress and inflammation during coronavirus infection, but also suppresses viral replication and improves antiviral immune defense and adrenal function [12,35].

Further, a meta-analysis found that vitamin D supplementation has protective effects against acute respiratory infections [36]. It might function through various mechanisms, such as maintaining intact epithelial layers, reducing the survival and replication of viruses, reducing the production of pro-inflammatory cytokines, and increasing ACE2 concentrations. According to a recent study [26], serum concentration should be raised above 40–60 ng/mL (100–150 nmol/L) for the treatment of COVID-19-infected patients [26]. The combined supplementation of vitamin D with melatonin could offer a reciprocal alternative for the prevention and treatment of pulmonary infection by COVID-19 [26,37].

Clinical studies [38] have shown that zinc supplementation can also reduce, by up to 54%, the severity and duration of various cold symptoms, such as fever, cough, sore throat, muscle pain and nasal congestion, which may also occur after SARS-CoV-2 infection. In a case-series study of four patients with COVID-19, the administration of high doses of oral zinc (up to 207 mg/day) was found to be possibly associated with improved oxygenation and fast resolution of shortness of breath after 1 day of treatment [38]. During an infection, an organism can mobilize zinc reserves for priority functions, such as those associated with the immune system, leading to a decrease in zinc levels and, possibly, to the lack of zinc for other less essential functions, such as the maintenance of smell and taste, senses often affected in patients with COVID-19 [38]. Previous scientific works strongly support our findings [35,36,37,38,39,40,41]. Further, this study was conducted before the start of mass vaccination against COVID-19 in Bangladesh. As a result, the findings are not affected by the administration of effective preventive measures such as vaccines.

To the best of our knowledge, this is one of the first studies to report the association of vitamins and micronutrients with the reduced risk of infection and reduced severity of COVID-19 in Bangladesh. Previous studies conducted on the impact of vitamin C, vitamin D and micronutrients lacked sufficient data collected directly from the participants. Further, studies assessing the roles of vitamin C, vitamin D and zinc in containing the infection were mainly on their roles in the immune system and clinical trials. Further, the previous studies found that both in the laboratory and real-life settings, vitamin C, vitamin D and zinc have an association with the reduction in COVID-19 infection and impact on the survival periods. Similar to previous studies, we also found that when the participants took only vitamins, they reduced the risk of severity of COVID-19. Further, this study found that both vitamins and medicines were also associated with a reduced risk of COVID-19.

The present study has a number of limitations. Firstly, the data are self-reported, and a larger sample could give more statistical power to the analysis. Further, in this study, we could not include contact tracing information. Though the data of different medicines such as analgesics, antivirals and antipyretics and vitamins were considered, the impact of vitamins on the outcome of the disease was analyzed separately in this study. Other factors including age, overall health conditions, genetic background, nutrition and lifestyle might have an impact on the overall outcome of COVID-19 infection. Further, the outcomes may vary from individual to individual based on these factors. In the future, controlled studies focusing on the impact of only vitamins can add more accurate knowledge. Moreover, adding information on cellular mechanisms and metabolic involvement can improve the understanding of the working principle and establish a definite role for vitamins and zinc supplements in the treatment of infectious diseases.

## 5. Conclusions

In conclusion, this study detected the significant association of vitamin C (ascorbic acid), vitamin D (cholecalciferol) and zinc (zinc sulfate monohydrate) supplementation in specific dosages with the outcome of COVID-19 in Bangladesh. However, we found that these vitamins and mineral were not significantly associated with infection rate and severity of the disease when they were taken through the diets of the patients as preventive measures or prophylactic options. The findings from this study suggest that vitamins and minerals with other prophylactics and preventive treatments might be associated with a lower risk of severe infection among patients. In the future, elaborate studies in controlled settings on the probable impact of vitamins and minerals in reducing the severity of COVID-19 should be conducted. Data from this study will provide baseline data for future studies for a more precise understanding of the contributions of vitamins and supplements in reducing cases and fatalities of COVID-19. Information from this study will add knowledge to improvement in the treatment options for COVID-19, which will ultimately aid in reducing cases and fatalities.

## Figures and Tables

**Table 1:** Socio-demographic characteristics of the participants.

Variables	Male	Female	Total
Study population	67.6% (650/962)	32.4% (312/962)	100.0% (962/962)
Age			
5–9	25.0% (1/4)	75.0% (3/4)	0.4% (4/962)
10–19	47.8% (22/46)	52.2% (24/46)	4.8% (46/962)
20–29	61.4% (239/389)	38.6% (150/389)	40.4% (389/962)
30–39	77.8% (144/185)	22.2% (41/185)	19.2% (185/962)
40–49	78.7% (122/155)	21.3% (33/155)	16.1% (155/962)
50–59	67.0% (71/106)	33.0% (35/106)	11.0% (106/962)
60–69	60.7% (37/61)	39.3% (24/61)	6.3% (61/962)
Above 70	87.5% (14/16)	12.5% (2/16)	1.7% (16/962)
Monthly income (Thousands in taka)			
Less than 10	54.0% (102/189)	46.0% (87/189)	19.6% (189/962)
10–29	73.2% (101/138)	26.8% (37/138)	14.3% (138/962)
30–49	80.6% (100/124)	19.4% (24/124)	12.9% (124/962)
50–79	76.0% (38/50)	24.0% (12/50)	5.2% (50/962)
More than 80	79.2% (19/24)	20.8% (5/24)	2.5% (24/962)
Not applicable	66.4% (290/437)	33.6% (147/437)	45.4% (437/962)
Residence			
Village	73.6% (39/53)	26.4% (14/53)	5.5% (53/962)
District town	70.4% (107/152)	29.6% (45/152)	15.8% (152/962)
Divisional city	66.6% (504/757)	33.4% (253/757)	78.7% (757/962)
Access to health services			
Less	73.6% (39/53)	26.4% (14/53)	5.5% (53/962)
Moderate	70.4% (107/152)	29.6% (45/152)	15.8% (152/962)
Better	66.6% (504/757)	33.4% (253/757)	78.7% (757/962)
Occupation			
Physician	57.9% (11/19)	42.1% (8/19)	2.0% (19/962)
Teacher	48.6% (18/37)	51.4% (19/37)	3.8% (37/962)
Researcher	100.0% (5/5)	0.0% (0/5)	0.5% (5/962)
Farmer	100.0% (2/2)	0.0% (0/2)	0.2% (2/962)
Nurse	24.1% (7/29)	75.9% (22/29)	3.0% (29/962)
Student	54.8% (176/321)	45.2% (145/321)	33.4% (321/962)
Journalist	100.0% (3/3)	0.0% (0/3)	0.3% (3/962)
Lawyer	0.0% (0/1)	100.0% (1/1)	0.1% (1/962)
Police	79.2% (19/24)	20.8% (5/24)	2.5% (24/962)
Banker	81.3% (26/32)	18.8% (6/32)	3.3% (32/962)
Administrative Officer	100.0% (7/7)	0.0% (0/7)	0.7% (7/962)
Private employee	96.1% (195/203)	3.9% (8/203)	21.1% (203/962)
Rickshaw driver/Van driver/Car driver	100.0% (24/24)	0.0% (0/24)	2.5% (24/962)
Businessman	94.2% (49/52)	5.8% (3/52)	5.4% (52/962)
Government employee	65.7% (23/35)	34.3% (12/35)	3.6% (35/962)
Others	50.6% (85/168)	49.4% (83/168)	17.5% (168/962)

**Table 2:** Frequency of uptake of vitamins, supplements and medicines by COVID-positive population.

Variables	Vitamin C		Vitamin D		Zinc		Medicines Taken for Treatment of COVID-19					
	Yes	No	Yes	No	Yes	No	Paracetamol 500 mg	Fexofenadine Hydrochloride 120 mg	Antibiotics	Montelukast Sodium 10 mg	Remdesivir 200 mg	None
Sex												
Male	52.7% (168/319)	47.3% (151/319)	52.7% (168/319)	47.3% (151/319)	48.6% (155/319)	51.4% (164/319)	74.6% (238/319)	48.3% (154/319)	59.9% (191/319)	37.0% (118/319)	11.3% (36/319)	9.1% (29/319)
Female	59.8% (110/184)	40.2% (74/184)	59.8% (110/184)	40.2% (74/184)	45.7% (84/184)	54.3% (100/184)	66.8% (123/184)	40.2% (74/184)	54.3% (100/184)	36.4% (67/184)	7.6% (14/184)	10.3% (19/184)
Age in years												
5–9	75.0% (3/4)	25.0% (1/4)	75.0% (3/4)	25.0% (1/4)	25.0% (1/4)	75.0% (3/4)	75.0% (3/4)	0.0% (0/4)	25.0% (1/4)	0.0% (0/4)	0.0% (0/4)	25.0% (1/4)
10–19	72.1% (31/43)	27.9% (12/43)	72.1% (31/43)	27.9% (12/43)	46.5% (20/43)	53.5% (23/43)	39.5% (17/43)	18.6% (8/43)	16.3% (7/43)	9.3% (4/43)	2.3% (1/43)	4.7% (2/43)
20–29	48.8% (83/170)	51.2% (87/170)	48.8% (83/170)	51.2% (87/170)	49.4% (84/170)	50.6% (86/170)	57.6% (98/170)	31.8% (54/170)	37.6% (64/170)	20.0% (34/170)	5.3% (9/170)	10.6% (18/170)
30–39	50.5% (52/103)	49.5% (51/103)	50.5% (52/103)	49.5% (51/103)	52.4% (54/103)	47.6% (49/103)	82.5% (85/)103	54.4% (56/103)	70.9% (73/103)	44.7% (46/103)	7.8% (8/103)	3.9% (4/103)
40–49	56.4% (44/78)	43.6% (34/78)	56.4% (44/78)	43.6% (34/78)	46.2% (36/78)	53.8% (42/78)	82.1% (64/78)	57.7% (45/78)	67.9% (53/78)	50.0% (39/78)	15.4% (12/78)	9.0% (7/78)
50–59	61.8% (34/55)	38.2% (21/55)	61.8% (34/55)	38.2% (21/55)	45.5% (25/55)	54.5% (30/55)	92.7% (51/55)	56.4% (31/55)	89.1% (49/55)	49.1% (27/55)	7.3% (4/55)	16.4% (9/55)
60–69	60.5% (26/43)	39.5% (17/43)	60.5% (26/43)	39.5% (17/43)	32.6% (14/43)	67.4% (29/43)	86.0% (37/43)	65.1% (28/43)	90.7% (39/43)	67.4% (29/43)	25.6% (11/43)	14.0% (6/43)
Above 70	71.4% (5/7)	28.6% (2/7)	71.4% (5/7)	28.6% (2/7)	71.4% (5/7)	28.6% (2/7)	85.7% (6/7)	85.7% (6/7)	71.4% (5/7)	85.7% (6/7)	71.4% (5/7)	14.3% (1/7)
Duration of supplementation												
7 days	22.3% (62/278)	-	22.3% (62/278)	-	18.4% (44/239)	-	19.9% (72/361)	20.2% (46/228)	17.5% (51/291)	11.9% (22/185)	16.0% (8/50)	29.2% (14/48)
14 days	34.2% (95/278)	-	34.2% (95/278)	-	28.0% (67/239)	-	43.2% (156/361)	39.9% (91/228)	39.5% (115/291)	42.7% (79/185)	26.0% (13/50)	37.5% (18/48)
21 days	10.4% (29/278)	-	10.4% (29/278)	-	18.0% (43/239)	-	14.4% (52/361)	15.8% (36/228)	15.5% (45/291)	16.2% (30/185)	22.0% (11/50)	8.3% (4/48)
1 month	21.2% (59/278)	-	21.2% (59/278)	-	22.6% (54/239)	-	18.0% (65/361)	18.0% (41/228)	19.6% (57/291)	22.7% (42/185)	20.0% (10/50)	10.4% (5/48)
2 months	5.4% (15/278)	-	5.4% (15/278)	-	5.9% (14/239)	-	1.9% (7/361)	3.1% (7/228)	3.4% (10/291)	2.2% (4/185)	6.0% (3/50)	6.3% (3/48)
More than 2 months	6.5% (18/278)	-	6.5% (18/278)	-	7.1% (17/239)	-	2.5% (9/361)	3.1% (7/228)	4.5% (13/291)	4.3% (8/185)	10.0% (5/50)	8.3% (4/48)
Times required to recover from the onset of the taking of vitamins and medicine												
1–5 days	45.9% (73/159)	54.1% (86/159)	45.9% (73/159)	54.1% (86/159)	42.1% (67/159)	57.9% (92/159)	67.3% (107/159)	45.9% (73/159)	51.6% (82/159)	28.3% (45/159)	6.9% (11/159)	7.5% (12/159)
7–14 days	55.7% (141/253)	44.3% (112/253)	55.7% (141/253)	44.3% (112/253)	47.8% (121/253)	52.2% (132/253)	77.1% (195/253)	44.3% (112/253)	59.3% (150/253)	38.3% (97/253)	10.3% (26/253)	9.1% (23/253)
15–30 days	68.1% (47/69)	31.9% (22/69)	68.1% (47/69)	31.9% (22/69)	56.5% (39/69)	43.5% (30/69)	63.8% (44/69)	44.9% (31/69)	69.6% (48/69)	46.4% (32/69)	10.1% (7/69)	13.0% (9/69)
No decrease	77.3% (17/22)	22.7% (5/22)	77.3% (17/22)	22.7% (5/22)	54.5% (12/22)	45.5% (10/22)	68.2% (15/22)	54.5% (12/22)	50.0% (11/22)	50.0% (11/22)	27.3% (6/22)	18.2% (4/22)
Symptoms												
Fever	52.8% (198/375)	47.2% (177/375)	52.8% (198/375)	47.2% (177/375)	38.7% (145/375)	61.3% (230/375)	81.9% (307/375)	47.7% (179/375)	63.7% (239/375)	43.5% (163/375)	10.4% (39/375)	4.0% (15/375)
Dry Cough	55.5% (101/182)	44.5% (81/182)	55.5% (101/182)	44.5% (81/182)	45.6% (83/182)	54.4% (99/182)	79.7% (145/182)	61.5% (112/182)	67.6% (123/182)	56.6% (103/182)	15.9% (29/182)	3.8% (7/182)
Loss of taste or smell	58.4% (101/173)	41.6% (72/173)	58.4% (101/173)	41.6% (72/173)	49.1% (85/173)	50.9% (88/173)	76.3% (132/173)	49.7% (86/173)	68.8% (119/173)	41.6% (72/173)	13.3% (23/173)	4.0% (7/173)
Fatigue	57.0% (65/114)	43.0% (49/114)	57.0% (65/114)	43.0% (49/114)	44.7% (51/114)	55.3% (63/114)	80.7% (92/114)	57.9% (66/114)	64.0% (73/114)	48.2% (55/114)	13.2% (15/114)	2.6% (3/114)
Body aches	54.7% (75/137)	45.3% (62/137)	54.7% (75/137)	45.3% (62/137)	40.1% (55/137)	59.9% (82/137)	82.5% (113/137)	59.9% (82/137)	63.5% (87/137)	43.1% (59/137)	11.7% (16/137)	2.9% (4/137)
Sore throat	68.2% (60/88)	31.8% (28/88)	68.2% (60/88)	31.8% (28/88)	60.2% (53/88)	39.8% (35/88)	76.1% (67/88)	53.4% (47/88)	63.6% (56/88)	53.4% (47/88)	18.2% (16/88)	1.1% (1/88)
Shortness of breath	59.2% (58/98)	40.8% (40/98)	59.2% (58/98)	40.8% (40/98)	40.8% (40/98)	59.2% (58/98)	80.6% (79/98)	58.2% (57/98)	62.2% (61/98)	70.4% (69/98)	26.5% (26/98)	1.0% (1/98)
Chest pain or pressure	54.1% (20/37)	45.9% (17/37)	54.1% (20/37)	45.9% (17/37)	54.1% (20/37)	45.9% (17/37)	78.4% (29/37)	51.4% (19/37)	54.1% (20/37)	59.5% (22/37)	29.7% (11/37)	2.7% (1/37)
Diarrhea	54.1% (20/37)	45.9% (17/37)	54.1% (20/37)	45.9% (17/37)	48.6% (18/37)	51.4% (19/37)	64.9% (24/37)	43.2% (16/37)	62.2% (23/37)	51.4% (19/37)	18.9% (7/37)	8.1% (3/37)
Loss of speech or movement	66.7% (16/24)	33.3% (8/24)	66.7% (16/24)	33.3% (8/24)	70.8% (17/24)	29.2% (7/24)	70.8% (17/24)	62.5% (15/24)	70.8% (17/24)	62.5% (15/24)	41.7% (10/24)	4.2% (1/24)
Inflammation of the eye	50.0% (2/4)	50.0% (2/4)	50.0% (2/4)	50.0% (2/4)	75.0% (3/4)	25.0% (1/4)	75.0% (3/4)	50.0% (2/4)	75.0% (3/4)	50.0% (2/4)	0.0% (0/4)	25.0% (1/4)
Rash	66.7% (2/3)	33.3% (1/3)	66.7% (2/3)	33.3% (1/3)	100.0% (3/3)	0.0% (0/3)	0.0% (0/3)	33.3% (1/3)	33.3% (1/3)	33.3% (1/3)	0.0% (0/3)	33.3% (1/3)
No symptoms	50.0% (2/4)	50.0% (2/4)	50.0% (2/4)	50.0% (2/4)	25.0% (1/4)	75.0% (3/4)	25.0% (1/4)	0.0% (0/4)	0.0% (0/4)	0.0% (0/4)	0.0% (0/4)	75.0% (3/4)
Duration of symptoms												
7–14 days	54.6% (191/350)	45.4% (159/350)	54.6% (191/350)	45.4% (159/350)	45.4% (159/350)	54.6% (191/350)	72.3% (253/350)	44.6% (156/350)	55.4% (194/350)	36.9% (129/350)	9.7% (34/350)	10.0% (35/350)
15–28 days	56.4% (75/133)	43.6% (58/133)	56.4% (75/133)	43.6% (58/133)	50.4% (67/133)	49.6% (66/133)	69.2% (92/133)	44.4% (59/133)	63.9% (85/133)	36.8% (49/133)	9.8% (13/133)	7.5% (10/133)
1–2 months	58.8% (10/17)	41.2% (7/17)	58.8% (10/17)	41.2% (7/17)	64.7% (11/17)	35.3% (6/17)	76.5% (13/17)	64.7% (11/17)	58.8% (10/17)	35.3% (6/17)	11.8% (2/17)	17.6% (3/17)
More than 2 months	66.7% (2/3)	33.3% (1/3)	66.7% (2/3)	33.3% (1/3)	66.7% (2/3)	33.3% (1/3)	100.0% (3/3)	66.7% (2/3)	66.7% (2/3)	33.3% (1/3)	33.3% (1/3)	0.0% (0/3)
Severity of symptoms												
No Symptoms	50.0% (2/4)	50.0% (2/4)	50.0% (2/4)	50.0% (2/4)	25.0% (1/4)	75.0% (3/4)	100.0% (4/4)	25.0% (1/4)	25.0% (1/4)	0.0% (0/4)	0.0% (0/4)	0.0% (0/4)
Mild Symptoms	53.9% (241/447)	46.1% (206/447)	53.9% (241/447)	46.1% (206/447)	46.3% (207/447)	53.7% (240/447)	70.0% (313/447)	44.7% (200/447)	56.6% (253/447)	35.6% (159/447)	7.6% (34/447)	10.7% (48/447)
Severe Symptoms	67.3% (35/52)	32.7% (17/52)	67.3% (35/52)	32.7% (17/52)	59.6% (31/52)	40.4% (21/52)	84.6% (44/52)	51.9% (27/52)	71.2% (37/52)	50.0% (26/52)	30.8% (16/52)	0.0% (0/52)
Outcome of the infection												
Death	36.4% (4/11)	36.4% (4/11)	36.4% (4/11)	63.6% (7/11)	45.5% (5/11	54.5% (6/11)	81.8% (9/11)	63.6% (7/11)	54.5% (6/11)	45.5% (5/11)	18.2% (2/11)	0.0%(0/11)
Recovery	55.1% (271/492)	44.9% (221/492)	55.1% (271/492)	44.9% (221/492)	52.6% (259/492)	47.4% (233/492)	71.5% (352/492)	44.9% (221/492)	57.9% (285/492)	36.6% (180/492)	9.8% (48/492)	9.8% (48/492)

**Table 3:** Association of socio-demographic factors with the uptake of vitamins and supplementation.

Variables	Medication	Vitamin C	Vitamin D	Zinc	Vitamin and Supplementation from Foods and Fruits
Age	0.002	0.04	0.03	0.05	0.02
Sex	0.05	0.02	0.04	0.21	0.34
Occupation	0.04	0.67	0.002	0.35	0.04
Residence	0.001	0.7	0.04	0.14	0.05
Monthly income	0.037	0.05	0.02	0.04	0.02
COVID-19 infection status	0.001	0.001	0.05	0.001	0.005
Symptoms	0.05	0.005	0.04	0.03	0.015

Association was determined by chi-square test, *p*-values < 0.05 was considered statistically significant.

**Table 4:** Impact of vitamins and different variables with infection rate of COVID-19 among participants.

Variables	OR (95% CI)	*p* Value
Age above 40 years	3.87 (1.91–5.84)	0.0001
Male	2.51 (1.21–4.67)	0.03
Taking vitamin C only as medication	0.34 (0.042–0.57)	0.003
Taking vitamin D only as medication	0.51 (0.014–0.76)	0.001
Taking zinc only as medication	0.72 (0.16–1.85)	0.005
Taking vitamin C and D as medication	0.04 (0.01–0.17)	0.00001
Taking vitamin C, D and zinc as medication	0.006 (0.03–0.11)	0.004
Increased eating of vitamin C-enriched foods	0.97 (0.51–1.85)	0.09
Increased eating of vitamin D-enriched foods	0.84 (0.01–0.17)	0.67
Taking supplements without medicine	0.02 (0.001–0.6)	0.02
Taking medicines without supplements	0.07 (0.01–0.94)	0.03
Taking both medicines and supplements	0.01 (0.006–0.09)	0.001
Taking only supplements as medication for 7 days or less	0.05 (0.01–0.67)	0.0007
Taking only supplements as medication for 7–14 days	0.001 (0.03–0.09)	0.0005
Taking only supplements as medication for more than 14 days	0.43 (0.14–0.97)	0.00005
No Symptoms	0.6 (0.23–1.73)	0.000001
Mild Symptoms	0.02 (0.03–0.6)	0.0001
Severe Symptoms	0.43 (0.1–0.9)	0.0004
Better access to health facilities	0.01 (0.001–0.2)	0.31
High income	1.8 (0.4–3.47)	0.0006
Symptoms prevailing >14 days	1.92 (0.57–4.28)	0.008
More than three symptoms	0.7 (0.24–1.8)	0.03
RT-qPCR confirmed cases	.03 (0.01–0.4)	0.00001
Non-confirmed suspected cases	0.07 (0.02–0.6)	0.0007

**Table 5:** Impact of vitamins and different variables on the development of the severity of COVID-19 among participants.

Variables	OR (95% CI)	*p* Value
Age above 40 years	5.61 (2.91–7.14)	0.05
Male	2.51 (1.21–4.67)	0.02
Taking vitamin C only	0.54 (0.01–0.92)	0.001
Taking vitamin D only	0.72 (0.3–0.98)	0.001
Taking zinc only	0.6 (0.11–1.2)	0.0001
Taking vitamin C and D	0.01 (0.001–0.09)	0.00001
Taking vitamin C, D and zinc	0.03 (0.01–0.22)	0.005
Increased eating of vitamin C-enriched foods	0.95 (0.72–2.52)	0.09
Increased eating of vitamin D-enriched foods	1.07 (0.68–2.9)	0.87
Taking supplements without medicine	0.8 (0.3–1.9)	0.005
Taking medicines without supplements	0.01 (0.008–0.07)	0.001
Taking both medicines and supplements	0.002 (0.001–0.009)	0.005
Taking only supplements for 7 days or less	0.6 (0.1–1.9)	0.54
Taking only supplements for 7–14 days	0.1 (0.01–0.8)	0.04
Taking only supplements for more than 14 days	0.23 (0.1–0.9)	0.05
Better access to health facilities	0.03 (0.01–0.6)	0.007
High income	0.4 (0.1–1.9)	0.001
More than three symptoms	3.9 (1.01–6.8)	0.05
RT-qPCR confirmed cases	1.9 (1.1–5.4)	0.001
Non-confirmed suspected cases	0.4 (0.2–0.96)	0.001

## Data Availability

Not applicable.
